# Neurofeedback Training Protocols in Sports: A Systematic Review of Recent Advances in Performance, Anxiety, and Emotional Regulation

**DOI:** 10.3390/brainsci14101036

**Published:** 2024-10-18

**Authors:** Beatrice Tosti, Stefano Corrado, Stefania Mancone, Tommaso Di Libero, Chiara Carissimo, Gianni Cerro, Angelo Rodio, Vernon Furtado da Silva, Danilo Reis Coimbra, Alexandro Andrade, Pierluigi Diotaiuti

**Affiliations:** 1 Department of Human Sciences, Society and Health, University of Cassino and Southern Lazio, 03043 Cassino, Italy; beatrice.tosti@unicas.it (B.T.); stefano.corrado@unicas.it (S.C.); s.mancone@unicas.it (S.M.); tommaso.dilibero@unicas.it (T.D.L.); a.rodio@unicas.it (A.R.); 2 Department of Medicine and Health Sciences “Vincenzo Tiberio”, University of Molise, 86100 Campobasso, Italy; chiara.carissimo@unimol.it (C.C.); gianni.cerro@unimol.it (G.C.); 3 Instituto de Psiquiatria-IPUB, Federal University of Rio de Janeiro, UFRJ, Rio de Janeiro 21941-853, Brazil; vernonfurtado2005@yahoo.com.br; 4 Faculty of Physical Education and Sports, Federal University of Juiz de Fora, UFJF, Juiz de Fora 36036-900, Brazil; daniloreiscoimbra@yahoo.com.br; 5 Health and Sports Science Center, Department of Physical Education, CEFID, Santa Catarina State University, Florianópolis 88035-901, Brazil; alexandro.andrade@udesc.br

**Keywords:** neurofeedback, EEG biofeedback, sports performance, cognitive performance, reaction time, emotional regulation, anxiety management, alpha-band training, beta-band training, athletic training

## Abstract

(1) Background. Neurofeedback has been used in sports since the 1990s, frequently showing positive outcomes in enhancing athletic performance. This systematic review provides an updated analysis of neurofeedback training in sports, evaluating reaction time, cognitive performance, and emotional regulation to address literature gaps and suggest future research directions. (2) Methods. A systematic search was conducted using PubMed, Scopus, Science Direct, and Web of Science databases for articles published from January 2016 to April 2023. The search included only original articles written in English, resulting in 24 studies meeting the inclusion criteria. (3) Results. The reviewed studies cover a wide range of sports, including golf, basketball, swimming, rifle shooting, football, volleyball, athletics, judo, ice hockey, triathlon, handball, fencing, taekwondo, and darts. They involved athletes of varying experience levels (beginners, professionals, and experts) and utilized neurofeedback training targeting different frequency bands (alpha, beta, theta, and SMR), either individually or in mixed protocols. Findings show improvements in sports and cognitive performance, emotional regulation, and anxiety management. (4) Conclusions. This systematic review supports the effectiveness of neurofeedback in enhancing sports and cognitive performance across various disciplines and experience levels. Notable improvements were observed in technical skills, physical performance parameters, scoring, attention, concentration, reaction time, short-term and working memory, self-regulation, and cognitive anxiety. Future research should standardize protocols, include more diverse samples, and explore long-term effects to further validate these findings.

## 1. Introduction

Neurofeedback is a non-invasive and almost side effects-free psychophysiological technique, built on the principles of operant conditioning [1]. However, it is important to note that while generally safe, some minor side effects may occur, such as headaches, fatigue, or dizziness, which are usually transient and resolve shortly after the session [2]. It uses changes in the brain’s electrical activity to help people regulate the power or activity of specific EEG frequency bands by real-time access to information related to their brain activity. Therefore, it has to do with real neurocognitive training by which the subject, thanks to the feedback immediately provided in a visual and/or auditory way, raises awareness of physiological states, changes their cerebral electrical activity, and corrects the EEG alterations and the dysfunctional states connected to them.

The activity of the brain can be measured using different signals acting as feedback, such as blood flow, oxygen consumption, and electrical activity. The latter, by means of EEG, represents the most commonly used form of neurofeedback [1]. Throughout the recording of the brain’s electrical activity, the EEG produces a trace in the form of cerebral waves, which provide data about brain functioning. These waves are traditionally split into five frequency bands (alpha, beta, gamma, theta, and delta), each represented by a specific range, and correspond to different cerebral states. The main advantages of EEG include its non-invasiveness, relatively low cost, and high temporal resolution, which allows for the detection of rapid changes in brain activity. However, EEG also has limitations, such as low spatial resolution, making it difficult to pinpoint the exact source of the electrical activity within the brain. Additionally, EEG signals can be contaminated by artifacts from muscle activity, eye movements, and external electrical interference [3,4].

Neurofeedback targets various brain regions depending on the specific goals of the training. For instance, the frontal lobes are often targeted for improving executive functions and attention, while the sensorimotor cortex is targeted for enhancing motor control and reducing anxiety. Different EEG frequency bands are associated with various states of brain activity:

Delta (1–4 Hz): Associated with deep sleep and unconscious states.

Theta (4–8 Hz): Linked to drowsiness, creativity, and meditative states.

Alpha (8–12 Hz): Related to relaxed wakefulness and a state of relaxed alertness.

Beta (12–30 Hz): Associated with active thinking, focus, and problem-solving.

Gamma (30–100 Hz): Related to high-level information processing and cognitive functioning.

Since its birth in the 1960s [5], neurofeedback has been used in different settings and with different goals: for example, as an alternative to pharmacological treatment in astronauts who were exposed to monomethyl hydrazine, a highly volatile rocket-fuel additive, and who suffered from headaches, nausea, and seizures [6,7]; as support in children afflicted with ADHD who showed an imbalanced brain wave pattern [8]; and as a tool to enhance performance, such as improving accuracy and speed in surgery skills [9], decreasing the number of errors in radar detection tasks [10], speeding up reaction times in attention tasks [11], and improving memory functions [12,13].

In addition to having proved effective in the treatment of these and other pathological and non-pathological conditions [14], neurofeedback has demonstrated the stability of its results over time [15,16,17]. Indeed, the neurophysiological changes induced by this technique are based on brain plasticity [18], and MRI studies have confirmed that these changes are associated with microstructural changes in white and grey matter [19], suggesting that neurofeedback may lead to enhanced cognitive processing and learning via improvement of the conduction velocity in neural networks.

Concerning the application of neurofeedback in improving performance, one line of research that has now taken hold concerns the use of neurofeedback in the field of sports psychology, in which this technique is used to rebalance brain-functioning patterns to improve sports performance in cognitive, emotional, and behavioral terms [20,21]. Its application in the field of sports dates back to the 1990s, when Landers et al. [22] exposed a group of archers to neurofeedback sessions and managed to improve their shooting performance.

Starting from this pioneering study, the enhancement of sports performance employing neurofeedback has become an increasingly investigated research field. Usually, the traits that define the quality of sports performance are reaction times [23], cognitive skills (attention, concentration, memory, inhibitory control, and focus) [24], perceptual-motor skills (such as passing accuracy and hand–eye coordination), and emotional states (such as anxiety and motivation) [25,26,27]; therefore, finding training procedures capable of enhancing these qualities is relevant. Effective neurofeedback training programs should seek to increase these components of performance. For example, neurofeedback has been shown to improve passing accuracy in rugby, demonstrating its efficacy in enhancing key perceptual-motor traits [20].

Specific neurofeedback (NF) training has proven to be effective, though not universally, for athletes in certain sports and at certain skill levels. For example, NF training has shown improvements in reaction times and cognitive performance in sports like swimming, judo, and golf, particularly among novice and elite athletes [20,21,28]. However, its effectiveness can vary based on the individual athlete’s baseline skill level and the specific sport. For example, Mikicin et al. [28] used EEG neurofeedback to amplify SensoriMotor Rhythm (SMR; 12–15 Hz) and beta1 bands (13–20 Hz) and to simultaneously reduce theta (4–7.5 Hz) and beta2 (20–30 Hz) bands in a sample consisting of swimmers, fencers, track and field athletes, judokas, and taekwondo athletes. They found that the training group showed more significant decreases in reaction times on a visual attention task than the control group and an increase in the speed, efficacy, and accuracy of performance. Like Mikicin’s study, Parsaee et al. [29] also investigated the effects of neurofeedback training on reaction times, in this case, both visual and auditory, and showed how this technique is actually effective at improving the brain functions associated with this kind of skill.

In the field of accuracy, Cheng et al. [30] showed that pre-élite golfers who underwent SMR neurofeedback training performed more accurately and exhibited greater SMR power than the control group, which is associated with an increase in attention, and Salimnejad et al. [20] found that the left and right passes’ accuracy in a sample of female rugby players increased significantly after neurofeedback training aimed at increasing SMR, whereas the shooting accuracy did not exhibit a significant improvement. With regard to cognitive and psychological performances, Liu et al. [24] showed that neurofeedback training proved to enhance the cognitive skills of athletes, resulting in an improvement in sustained attention ability.

While most studies reviewed support the effectiveness of neurofeedback in enhancing sports and cognitive performance, there are notable exceptions. For instance, Mirifar et al. [31] found no improvements in attention and reaction time following NF training aimed at decreasing theta and beta bands. Similarly, Dupee [32] reported no changes in athletes’ scores despite improvements in physiological and psychological conditions. These discrepancies highlight that NF may not be universally effective and its benefits can depend on various factors, including the specific sport, the protocol used, and the individual characteristics of the athletes.

Hence, to provide an updated and comprehensive review of the latest developments in neurofeedback training in sports disciplines, this study reviews articles published between 2016 and 2023, including both randomized and non-randomized studies. The primary objectives of this review are to categorize published neurofeedback-related articles from the perspectives of reaction time, cognitive performance, and perceptual-motor skills, analyze and evaluate these studies to fill gaps in the neurofeedback sports-related literature, and suggest directions for future research.

The rationale for focusing on articles published between 2016 and 2023 lies in capturing the most recent advancements and trends in neurofeedback research within the realm of sports. This timeframe ensures that the review encompasses contemporary studies that reflect the latest technological and methodological innovations, thereby providing an up-to-date perspective on the efficacy and application of neurofeedback in enhancing athletic performance. By including both randomized and non-randomized studies, the review aims to offer a comprehensive overview that acknowledges various research designs and their respective contributions to the field. The inclusion criteria are designed to ensure the selection of high-quality, empirical studies that specifically measure the effects of neurofeedback on sports and cognitive performance, thereby filling gaps in the existing literature and guiding future research directions.

In this review, we distinguish between cognitive performance and athletic performance. Cognitive performance refers to mental abilities measured through tasks like the digit span test and working memory assessments, whereas athletic performance pertains to physical skills directly related to sports, such as reaction time and motor control. While both types of performance are important for athletes, we acknowledge that cognitive tasks do not directly measure sports-related outcomes but are crucial in enhancing overall performance in sports contexts. Therefore, we included studies that evaluated both cognitive and athletic performance in athletes to provide a more comprehensive understanding of neurofeedback’s effects.

## 2. Materials and Methods

This systematic review adhered to the Preferred Reporting Items for Systematic Reviews and Meta-Analyses (PRISMA) guidelines [33].

### 2.1. Research Strategy

The following four electronic bibliographic databases were used to carry out this review: PubMed, Scopus, Science Direct, and Web of Science. Only studies published between January 2016 and April 2023 were selected. The literature search was conducted over five days from 16 April to 20 April 2023, with an additional final search conducted in May 2023. To ensure comprehensive coverage of relevant studies, a wide range of search terms was employed using the Boolean operators “AND” and “OR”. The search terms included but were not limited to “neurofeedback”, “EEG biofeedback”, “neural feedback”, “sport*”, “athlete”, “perform*”, “EEG”, “biofeedback”, “cogniti*”, “reaction time”, “response time”.

The search syntax was designed to capture variations of key terms (e.g., sport*, perform*, cogniti*) to include studies with terms such as sports, performance, cognition, etc. This approach aimed to avoid the omission of relevant studies due to specific wording. The search was performed by one author and included article titles, abstracts, keywords, and publication years.

All electronic search results were imported into Rayyan software for analysis [34]. The screening, eligibility, and selection of studies followed a three-step process. First, duplicate records were removed. Second, based on title and abstract screening, publications that were clearly irrelevant to the review topic were excluded. Finally, the full texts of potentially relevant studies were retrieved and examined for eligibility.

### 2.2. Selection of Studies

The screening, eligibility, and selection of studies were carried out by one author (B.T.). The selected studies were analyzed by Rayyan software [34]. The choice of eligible studies was supported by a three-step procedure. The first step consisted of merging all the records and removing the duplicate ones. Secondly, results considered not relevant to the topic were also excluded from the analysis. As the third and last step, one author (S.C.) examined all remaining publications for eligibility (see the inclusion and exclusion criteria below).

### 2.3. Inclusion Criteria

The requirements needed to include publications in this review are the following: (1) written in English; (2) published between January 2016 and April 2023; (3) include pre- and post-intervention assessments; (4) have been carried out on healthy sportspeople (no age range was set for this review); (5) original, empirical articles; (6) either a randomized controlled trial (RCT) or a non-randomized controlled study (NRS); (7) either published or in press articles; and (8) measure the effects of a neurofeedback training on sports and cognitive performance of athletes.

### 2.4. Exclusion Criteria

The studies excluded in the review were characterized by one or more of the following features: (1) carried out on subjects suffering from physical, neurological, and/or psychiatric diseases or undergoing pharmacological treatment; (2) a complete report of their methods (especially the location of electrodes and the selected frequency targeted by neurofeedback) was missing; and (3) qualitative studies, narrative or systematic reviews, meta-analyses, book chapters, and conference papers.

### 2.5. Data Extraction

Data extraction was performed by one author (B.T.) and audited for accuracy and completeness by a second author (S.C.). The information obtained from each study was recorded in an Excel sheet and included publication details (authors and year), population characteristics (number of participants, gender, age, sports discipline, and expertise level), and study characteristics (study design and procedure, neurofeedback device, frequency and duration of the training sessions, electrode position, intervention, feedback used, control group, outcomes, and intervention effects). Information on the selected studies is presented in Table 1.

### 2.6. Risk of Bias Assessment

We used the Mixed-Methods Appraisal Tool (MMAT, version 2018 [60]) to investigate possible sources of bias. This checklist has been used effectively in other systematic reviews in the field of sports psychology [61,62,63] and permits researchers to appraise the methodological quality of five different types of study design: qualitative studies, quantitative descriptive studies, randomized controlled trials, non-randomized quantitative studies, and mixed-methods studies, and comprises up to five methodological criteria for each of them, rated on a nominal scale (yes, no, cannot tell). Articles were rated by one author (B.T.) and checked for accuracy by a second author (S.C.). In particular, studies employed for this work are divided into randomized controlled trials, non-randomized controlled trials, and non-controlled trials. For randomized controlled trials, we checked the appropriateness of the randomization, the comparison level of groups concerning baseline, the completeness of the data, the execution of the blinding operation, and if participants adhered to the assigned intervention, with the potential total score going from 20% (meeting one criterion only) to 100% (all five criteria met). For non-randomized studies, we assessed methodological quality by analyzing if the sample was representative of the target population, if measurements were appropriate, if outcome data were complete, if confounders were accounted for, and if the intervention was administered as intended, with the potential total score ranging from 20% (one criterion met) to 100% (all five criteria met). Importantly, to ensure transparency and allow readers to assess the quality of these studies, the scores and comments for each article reviewed are reported, although no studies were excluded based on the assessment of methodological quality (see Table 2).

## 3. Results

### 3.1. Study Selection

**Figure 1:**
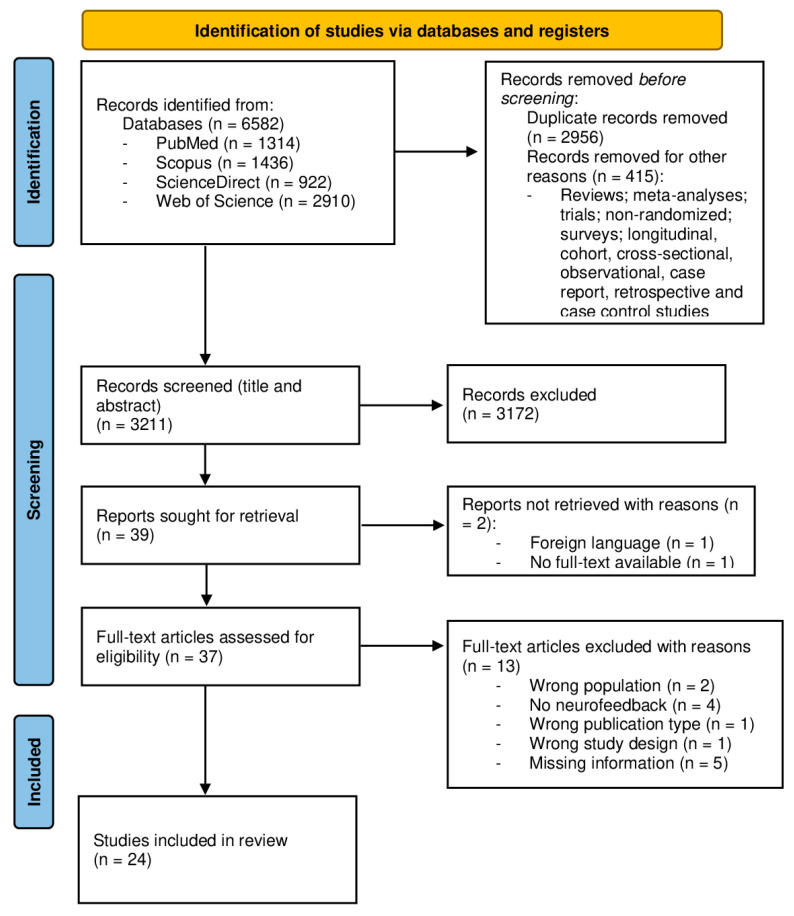
PRISMA (Preferred Reporting Items for Systemic Reviews and Meta-Analysis) flow diagram of search strategy.

The research strategy showed 6582 potentially relevant studies. The retrieved articles were screened in three stages: first, all the duplicates (2956 articles) were removed, and another 415 studies were excluded because of wrong publication types and wrong study designs; thereafter, based on the screening of titles and abstracts, publications that were clearly irrelevant to the topic under review, despite mentioning the search terms, were excluded (3172 articles). For these two steps, we used Rayyan software [34]. At this point, 39 articles met the eligibility criteria, so we sought them for retrieval. Two articles were additionally excluded, one due to the impossibility of retrieving the full text and another one because it did not meet the language eligibility criterion. Finally, the full texts of the remaining 37 studies were downloaded and read and 13 articles were not included. The reasons for the exclusion were as follows: missing information about the electrode location and the frequency band(s) targeted by neurofeedback (5 articles), neurofeedback not carried out on sportspeople (2 articles), studies sought for retrieval because they mentioned feedback training in the title and/or abstract but did not use an EEG-neurofeedback training (4 articles), wrong publication type (1 review article), and wrong study design (1 qualitative study). At the end of the selection process, 24 articles were included in this review. The results of the literature search are presented in the PRISMA flow diagram below (Figure 1).

### 3.2. Study Characteristics

**Figure 2:**
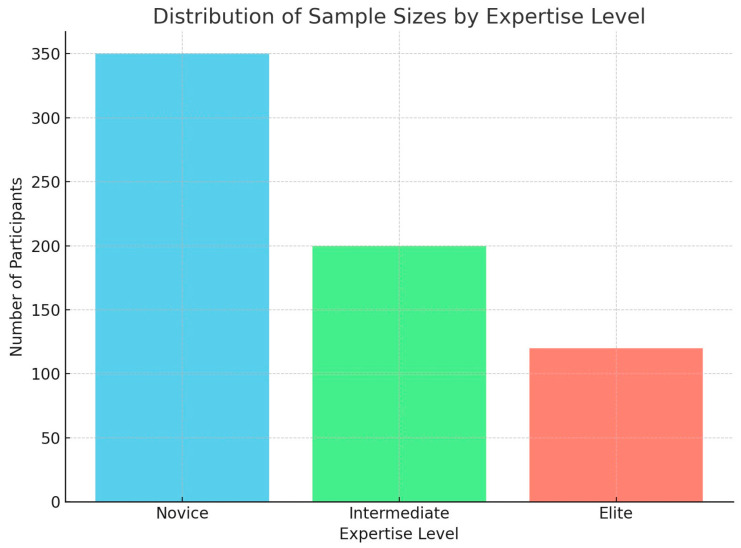
Distribution of sample sizes by expertise level.

Table 1 summarizes the main characteristics of the selected studies. Concerning the sports discipline, two studies investigated soccer [31,35], two track and field [35,58], one volleyball [37], three judo [39,40,41], two swimming [45,58], one basketball [50], one triathlon [56], two shooting [40,46], one darts [41], one handball [42], one ice hockey [47], one cycling [54], three golf [55,57,59], and five studies did not specify which type of sport they examined [44,49,51,52,53]. Two studies [35,58] reported data from two different sports (soccer and track and field, and swimming and track and field, respectively). For the number of participants, the total sample of the 24 studies selected for this review included 746 participants, of which 55 were non-athletes (reported by Domingos et al. [49] and by Kober et al. [56] and 691 were sportspeople. The athletes’ level included 47 professional athletes [35,40,42], 34 élite athletes [35,37,42], 15 non-élite athletes [37], 76 student/University level athletes [46,47], 24 international level athletes [39,48], and 145 novice athletes [41,50,55,59]. Twelve studies did not report information about the level of the remaining 350 athletes [31,39,44,45,49,51,52,53,54,56,57,58]. As shown in Figure 2 below, the distribution of sample sizes indicates that novice athletes were most frequently represented, followed by intermediate and elite athletes.

There was a total of 129 women: 12 non-athletes included in the control group by Kober et al. [56] and 117 sportswomen split into 9 élite athletes [42], 18 University level athletes [47], and 35 novice athletes [55,59]. The level of the remaining 55 women athletes was not specified [51,52,54,56], and 6 articles did not report if they included women in their studies [35,39,45,49,52,58].

The mean age ranged from 12.87 years for the participants in the study by Dana et al. [44] to 37.1 years for the athletes in the study by Chen et al. [57].

With regard to the neurofeedback device, seven studies used ProComp Infiniti + BioGraph Infinity software [37,43,44,47,50,57], one study used a neurofeedback system for home training [35], one study used the Enobio wireless EEG monitoring device and the Neuroelectrics Instrument Controller, v 1.1 − NIC 1.1 + Biograph Infiniti software [39], two studies used the EEG DigiTrack Biofeedback System [40,42], one study used the Nexus-10 MKII system + BioTrace software [31], two studies used System Flex 30 + TruScan software [28,45], one study used the Deymed TruScan System (software version 6.34.1761 [48]), one study used the EEG training plugin included in the Somnium software [51], three studies only reported the software they used (BioExplorer software [56]; BioTrace software [55]; SIMULINK software [56]), and five studies did not mention either the device or software used [21,41,51,52].

Eleven studies trained the alpha power, 10 trained the beta band, 9 trained the theta frequency, and 10 trained the SMR rhythm; also, 10 studies trained more than one frequency band at the same time (see Table 1). The majority (12) of the studies included in this review used visual feedback, 5 studies used auditory feedback, and 7 used a combination of both. Furthermore, the intervention period ranged from 1 day to 4.5 months, and the daily neurofeedback duration ranged from 4 min to 1.5 h (see Table 1).

### 3.3. Risk of Bias in Studies

All studies included in this review were assessed for reporting quality based on the standards of the MMAT (version 2018) [60]. Seventeen studies used a randomized controlled design, while the remaining seven were quantitative non-randomized studies. Of the randomized controlled studies, one (5.88%) scored 20%, seven (41.18%) scored 40%, eight (47.06%) scored 60%, and one (5.88%) was judged to be of high quality (80%), while with regard to the non-randomized studies, one (14.28%) scored 40%, five (71.43%) scored 60%, and one (14.28%) was judged to be of high quality (80%). These two groups of studies were evaluated using five criteria each. Going into the specifics of the randomized controlled trials, regarding selection bias, 14 studies (82.35%) did not report how randomization was performed for assigning participants to each group. Taking the detection bias into account, only 4 of the studies (23.53%) stated that the outcome assessors were blinded to the intervention provided, while the other 13 studies (76.47%) did not provide any information regarding this criterion. Furthermore, in the area of attrition bias, four studies (23.53%) did not report complete outcome data for different reasons (poor-quality data, participant injury, and dropout). Finally, in 7 out of 17 studies (41.18%), groups were comparable at baseline, and only in 1 study did participants not completely adhere to the assigned intervention as they completed fewer neurofeedback sessions than required by the study in which they participated. As for the non-randomized studies, none of them (0%) met the criterion of representativeness of the sample either because they lacked clear-cut points for the inclusion of participants or because of the sampling method used (convenience or consecutive sampling), as a result of which all studies showed selection bias. The second characteristic assessed was the appropriateness of the measures regarding both the outcome and intervention, and all included studies (100%) fulfilled this criterion. With regard to the detection bias, only two studies (28.57%) did not report complete outcome data due to insufficient EEG signal quality or contamination by artifacts in the EEG epochs. Taking confounders into account, these were only mentioned in four studies (57.14%). Finally, the last characteristic assessed was the administration of the intervention, and the results of the MMAT showed that only one study did not fulfill this requirement, as participants underwent the intervention less than required. As previously reported (see the “Quality Assessment” section), no studies with low methodological quality were excluded since it is not recommended [60], hence Table 2 shows the evaluation of each criterion taken into account for each study in order to provide a better illustration of the quality of the included studies.

### 3.4. Synthesis of Results

The dependent variables examined in this review relate to the sports performance (score, physical performance, physical parameters, and technical aspects important for performance) and cognitive performance (in terms of reaction time, self-regulation, attention, concentration, memory, and stress) of athletes. With regard to randomized controlled trials, 16 out of 17 showed a positive effect of neurofeedback on sports and cognitive performance, while only the study by Mirifar et al. [31] did not show improvements in attention and reaction time following neurofeedback training aimed at decreasing theta and beta bands in one group and increasing SMR rhythm in another. Eight studies considered the variable “sports performance” [39,41,46,47,50,55,56,59] using neurofeedback training targeting different frequency bands: alpha (five studies) [21,41,50,54,55], theta (three studies) [39,47,50], beta (two studies) [39,47], and SMR (four studies) [21,47,50,57].

Some studies used protocols that involved acting on several frequency bands at the same time or compared experimental groups submitted to different brainwave training and therefore were counted more than once. These studies showed an improvement in performance induced by neurofeedback in the following considered components of sports performance: technical aspects important for performance [39,41,54,55] and scoring [21,47,50,59]. Nine studies considered the variable “cognitive performance” [40,41,43,48,49,51,52,53,56] using beta waves (three studies) [40,43,48], alpha (five studies) [41,49,51,52,53], theta (two studies) [43,48], and SMR waves (two studies) [43,56] as neurofeedback targets and observed an improvement in attention and reaction time in the COG Test and Oddball Task [40,43,48,49,51,52], short-term memory in the Digit Span Test [49,52], working memory in the N-Back Test [51,52], self-regulation [56], cognitive anxiety [41], and HRV, which has been related to cognitive performance, such as information processing, attention regulation, anxiety, and stress [53]. Furthermore, the study by Domingos et al. [51] submitted two groups of athletes to neurofeedback sessions in silent and noisy conditions and showed that the group exposed to intermittent noise obtained positive results both in the working memory test (*p* = 0.005) and the reaction time test (*p* = 0.003).

As far as non-randomized studies are concerned, all studies showed significant effects of neurofeedback in improving sports and cognitive performance of athletes. In particular, four studies examined the variable “sports performance” [28,37,44,57] using neurofeedback training targeting different brain waves: SMR (two studies) [37,44], alpha (one study [37], beta (two studies) [44,58], and theta (two studies) [44,57]. Again, some studies were counted more than once as they examined different frequency bands. These studies showed an improvement in performance, induced by neurofeedback, in the following components of sports performance: scoring [37,57] and physical parameters important for performance [44,58].

Six studies took into consideration the variable “cognitive performance” [35,37,42,44,45,58] using alpha (two studies) [35,37], beta (four studies) [42,44,45,58], theta (two studies) [42,44], and SMR waves (two studies) [37,42,44] as neurofeedback targets and observed an improvement in the RESTQ (Recovery and Stress Questionnaire score [35], a reduction in the use of self-talk [37], and an improvement in concentration and attention [42,58], working memory in the Wechsler Digit Span Test [44], and mental work performance in the Kreapelin Test [45].

**Figure 3:**
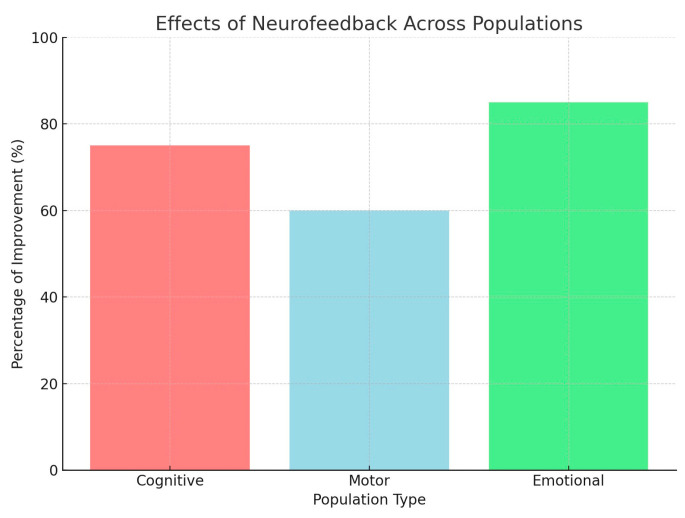
Effect of neurofeedback across populations.

Figure 3 below visualizes the improvements in cognitive, motor, and emotional regulation observed in the reviewed studies, showing the highest percentage of improvement in emotional regulation.

### 3.5. Practical Implications of Neurofeedback across Different Sports

**Figure 4:**
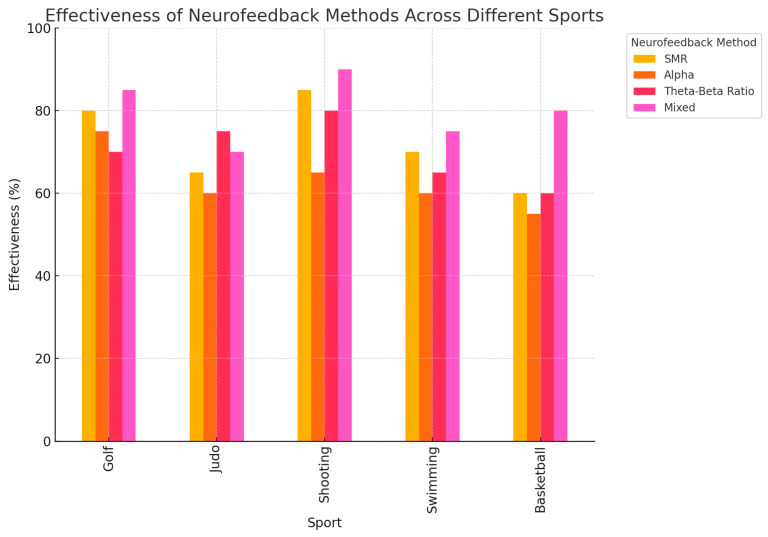
Effectiveness of neurofeedback methods across different sports.

The efficacy of neurofeedback varies according to both the sport and the specific cognitive or motor skills targeted. For example, SMR training has proven particularly effective in improving accuracy in sports such as golf and rifle shooting, where fine motor control and sustained attention are critical [30]. In contrast, training focused on beta waves has been shown to enhance dynamic balance and cognitive performance in sports like judo and swimming, where quick decision-making and reaction times are essential [39]. Therefore, while neurofeedback shows general benefits across various sports, the choice of frequency band and protocol should be tailored to the sport’s specific demands. Alpha training, for example, is beneficial for athletes in sports requiring high levels of visuospatial skills and relaxation, such as darts and archery [41]. As shown in Figure 4, SMR training was particularly effective in precision-based sports like shooting and golf, while the mixed protocol yielded the highest improvements across multiple sports disciplines.

### 3.6. Impact of Expertise

The results of this review suggest that the level of athlete expertise significantly influences the outcomes of neurofeedback training. The studies reviewed involved athletes at various levels, from novices to experts and professionals. For instance, novice athletes exhibited notable improvements in fundamental skills such as motor control and reaction times, while elite athletes benefited from more specific and subtle improvements, such as enhanced focus and anxiety management. These findings indicate that neurofeedback effectiveness may be affected by the initial skill level, highlighting the need for differentiated protocols based on the athlete’s experience.

## 4. Discussion

The purpose of this systematic review is to provide an updated overview regarding the latest developments in the use of neurofeedback technique in the field of sports psychology, with particular reference to the effects on sports performance (improvement in scores and physical and technical parameters important for successful performance) and cognitive performance (improvement in attentional and memory skills, concentration, reaction time, self-regulation, and stress management) of athletes. The results of this review indicate that a significant number of studies support the effectiveness of neurofeedback in improving athletes’ sports and cognitive performance. However, it is important to note that there are exceptions, such as the 2019 study by Mirifar and colleagues [31], which did not find improvements.

Advances in neurofeedback training (NFT) have been shown to significantly impact three key domains in athletes: performance, anxiety, and emotion regulation. NFT has demonstrated notable performance improvements in sports requiring precision and fine motor skills, such as shooting, darts, and golf. In these sports, NFT primarily targets brainwaves associated with focus and accuracy, such as the suppression of theta and enhancement of SMR waves. Athletes in these disciplines showed improved reaction times and coordination, likely due to the role of SMR in maintaining sensorimotor rhythm, which is crucial for precision tasks. In team sports such as soccer, volleyball, and handball, where athletes are often subjected to high-pressure environments, NFT helped reduce anxiety by increasing alpha wave activity and reducing beta2 activity. The observed reduction in anxiety might be due to the impact of neurofeedback on enhancing athletes’ ability to regulate stress, especially in competitive scenarios. The distinction between the nature of team sports and individual precision sports suggests that NFT may need to target different frequency bands depending on the psychological demands of the sport. Sports involving endurance or prolonged focus, such as swimming and triathlon, benefitted from NFT’s ability to regulate emotions. By increasing SMR and alpha activity, NFT helps athletes maintain emotional stability, which is essential for sustaining long periods of physical and mental exertion. Emotion regulation improvements may also stem from the athletes’ enhanced ability to manage stress and maintain a calm, focused state during competition. Overall, the differential effects of NFT across sports suggest that specific neurofeedback protocols may be more effective depending on the cognitive and psychological demands of each sport. Future research should explore the customization of NFT protocols to maximize benefits in these distinct domains.

The frequency bands trained and the protocols used are diverse, and while many suggest positive effects, the outcomes can vary depending on several factors, including the specific application and individual differences among athletes. As also demonstrated by the studies included in this review, cognitive and sports performance are very often interrelated, with the enhancement of one being closely related to the enhancement of another. Therefore, we will not discuss these aspects separately but rather will try to integrate them starting from the role that each brain wave plays in the individual’s cognitive functioning.

In the studies included in this review, different neurofeedback protocols were used, focusing on training the alpha, theta, and beta bands and the SMR rhythm. Relative to the alpha band, all studies showed significant effects on sports performance, some of which used a protocol aimed exclusively at this frequency [41,54,55], while others used mixed protocols [37,46,50]. Alpha band regards spatial attention to visual targets and visuospatial information processing [64], information processing speed [65], mnemonic functions [66,67], and reaction time [68].

The study by Norouzi et al. [41] showed how Quiet Mind Training (consisting of alpha rhythm suppression) contributes to the improvement of implicit motor learning in novice athletes, leading precisely to an increase in visuospatial resources, which, in turn, results in improved motor performance. It is important to note that some studies, such as the one conducted on novice dart players, have shown promising results with neurofeedback training. However, these findings may not be directly generalizable to athletes in other sports or to those with different levels of expertise. This specific result applies to the population of novice dart players, and further research is needed to determine its applicability across a wider range of sports and skill levels.

Moreover, Norouzi et al. [41] showed that motor performance was not affected by stress conditions, confirming what has already been shown by Masters [69], Lam et al. [70], and Vine et al. [71]. According to the latter, since cognitive overload and psychological pressure disrupt implicit motor skill processing, stress down-regulation can promote implicit learning techniques and, therefore, unconscious control. However, an alternative explanation could stem from improved concentration or reduced anxiety and stress through Quiet Mind Training, as it is capable of generating the so-called “flow state”, a mental state associated with reduced conscious attention and increased safety, calm, focus, and automaticity [72].

In line with the approach–withdrawal model of frontal asymmetry, which links left frontal activity to processes related to approaches and right frontal activity to avoidance-related processes, frontal alpha activity has also been connected to emotional and motivational processes [73,74]. Mottola and colleagues [54] first investigated the effects of neurofeedback on endurance performance, demonstrating that increasing left frontal cortical (NFL) activity has a positive effect on this type of performance as it supports participants to exercise for longer periods of time while experiencing a high level of physical exertion and helps them maintain focus and involvement in the increasingly painful and strenuous task, thereby delaying their need to stop and retreat.

A well-known component of the alpha frequency band is the Mu rhythm, which reflects the allocation of cognitive resources to respond to motor programming [75] during the execution of goal-directed actions [76], and in studies of sports, a decreased Mu pace has been associated with increased performance in golf putting [77], success in the putting task (i.e., the number of balls put in the hole [78,79,80]), and action correction [81]. In line with these findings, the study by Wang et al. [55] showed that a decrease in Mu rhythm leads to improved motor performance in complex visuomotor skills, such as golf putting, after a single neurofeedback session. The improvement in motor performance could be due to a joint action of visuomotor performance, Mu rhythm, and the level of attentional control of action (as Mu rhythm is strictly connected to motor control). Such elements allow for inferring that there is a kind of inverse proportionality between the Mu rhythm and the allocation of cognitive resources to respond to motor programming. Such allocation turns into adaptive motor control and increased levels of action control during complex visuomotor tasks [82,83] and thus, consequently, improves performance.

Consistent with the cognitive functions covered by the alpha frequency band, the study by Domingos et al. [49] showed an improvement in reaction times (Oddball Task), confirming the results obtained by Klimesch in 1999 [84]. In addition, in 2021, Domingos and colleagues [51,52,53] conducted a series of studies showing that a noisy environment had positive effects on tests of working memory (N-Back) and reaction times (Oddball Task), that cognitive performance in these same tasks was better if three sessions of neurofeedback per week were performed compared to two sessions per week (suggesting that a concentrated training protocol leads to better results), and that a protocol consisting of three workouts per week led to an improvement in Heart Rate Variability (HRV), supporting the previous study by Alexeeva et al. [85]. HRV is a primary figure of merit in the sports field as it has been linked to Autonomic Nervous System function, cardiovascular control [86], and cognitive performance (information processing, attention regulation, anxiety, and stress) [87,88,89,90,91].

The study by Rijken and colleagues [35] showed how a peak performance program along with neurofeedback training leads to changes in performance and stress reduction, supporting the study by Dekker et al. [92], in which alpha training conducted on gymnasts showed changes in sleep quality and physical and mental fitness. Although several studies included in this review suggest that neurofeedback may aid in stress regulation, the interaction between stress and performance is complex. Research on acute stress indicates that its effects on both psychological and physiological performance are multifaceted [93,94]. While neurofeedback shows promise in helping athletes manage stress, it is important to acknowledge the variability in individual responses to stress and the specific stressors involved. Therefore, more research is needed to explore the effectiveness of neurofeedback in high-stress competitive environments and its impact on both cognitive and athletic performance.

The SMR wave corresponds to the frequency band of the sensorimotor cortex and shows an inverse correlation with sensorimotor cortex activity [95]. This suggests that reduced thalamic activity is associated with decreased interference in somatosensory processing [92]. Therefore, higher SMR rhythm power corresponds to a mental state of neural processing during psychomotor and attentional tasks [96,97]. In the context of sports disciplines, some research has shown that a high SMR rhythm in the final stage of motor preparation is associated with better performance in darts shooting [98], golf putting [30], and a firearm shooting task [97], suggesting that a high SMR rhythm may be an indicator of greater psychomotor efficiency during movement execution. Based on this evidence, Christie et al. [47] demonstrated the ability of SMR training to improve sports performance in ice hockey players. The interesting aspect of this study is that although participants were shown to be able to increase their SMR rate in the laboratory setting, they were not able to do the same during field hockey shooting performance.

A number of studies [99,100] reported that prior to movement, the alpha and beta bands desynchronize on the sensorimotor cortex, and this may be attributed to motor preparation and execution. Further confirmation comes from the 2019 study by Christie et al. [101] who showed that the SMR rhythm desynchronizes before the illumination of a target light. Therefore, participants’ inability to increase the SMR rhythm during performance could be due to either their inability to transfer learning from the laboratory to the performance condition or an event-related SMR desynchronization (i.e., a reduction in amplitude) that occurs during motor preparation and execution [102].

Another study that used neurofeedback training to target the SMR wave is that of Pourbehbahani et al. [59] in which it was shown that SMR rhythm enhancement leads to an improvement in golf putting performance, working to confirm the results obtained by other studies [21,30,103], and this improvement could be due to the facilitation of motor learning through the suppression of motor and cognitive processes irrelevant to the task [77], consistent with the psychomotor efficiency hypothesis [104]. Ultimately, research by Kober and colleagues [56] on triathletes showed how they were able to self-regulate their brain activity, meaning that they outperformed the control group in training by sustaining the mental state required to improve SMR pace power for an extended amount of time. This supports the assumption that athletes are generally more trained in self-regulation and the ability to ignore task-irrelevant thoughts, which is important for the self-regulation of physical activity and also brain activity [18,105,106,107,108,109,110,111,112,113,114,115,116,117]. Moreover, triathletes exhibited augmented bilateral white matter volume in the inferior frontal gyrus, insula, and orbitofrontal cortex. The existing literature suggests that heightened physical activity results in expanded volumes in these areas, which are associated with enhanced cognitive control abilities facilitating effective self-regulation of physical activity [118,119,120,121,122,123].

The insula and the medial and inferior frontal gyri are related to interoceptive perception and focused attention on interoceptive states [124]. These cognitive processes play a pivotal role in the autoregulation of brain activity [18,107,110,113,125]. As stated by Hatfield et al. [126] and Wulf [127], internal attentional focus may inhibit “automatic” behavior and decrease performance quality. However, triathletes could potentially be more efficient in focusing attention on their internal states while ignoring irrelevant stimuli and thoughts, which could lead to an improvement in neurofeedback performance. In light of the positive results from the study by Kober et al. [96] with triathletes, further discussion on this topic could be highly interesting and beneficial.

Based on the functions that alpha and SMR waves play in relation to sports performance, three of the studies we examined used a neurofeedback protocol aimed at training both frequency bands. Hosseini and Norouzi [37] showed a reduction in self-talk and improvement in service scores in elite and non-elite volleyball players due to a reduction in distractions achieved through alpha wave training. In their study, Gong et al. [46] observed a significant improvement in shooting performance in the SMR group and a decline in performance in the alpha group. This decline could be due to the fact that, although the alpha group was tasked with increasing the alpha rhythm in the left temporal region and decreasing it in the right temporal region, the results may have led to increased activity in both brain hemispheres because the participants did not acquire their shooting skills through training and performance did not improve.

Shokri and Nosratabadi’s investigation [50], which involved basketball players, is another study that shows the efficacy of an alpha–SMR neurofeedback intervention in conjunction with a biofeedback intervention. The researchers hypothesized that, in the group receiving the combined intervention, neurofeedback may have improved performance (in lay-up, chest passing, dribbling, and free-throw shooting) by increasing attention and alertness and reducing reaction time, important components in each of the four performance indicators assessed [128,129,130,131,132,133].

Another widely used neurofeedback protocol is the “theta–beta ratio” (TBR), which is the ratio of theta-band to beta-band activity, consisting of suppression of the theta rhythm and enhancement of the beta frequency. It has been observed that the TBR is related to decision making [134,135,136], attentional control [137,138,139,140], and the down-regulation of negative feelings [141], so its application could be effective in relation to various phenomena and conditions, including improving performance in sports.

In this regard, Maszczyk et al. [39] showed how a reduction in the values of theta and alpha waves, along with an increase in the values of beta rhythm, leads to a significant improvement in the dynamic balance of judokas from the sixth/seventh session of intervention, supporting the results obtained by Hammond in his 2005 studies [142,143]. In addition, Dana et al.’s study [44] of 10- to 14-year-old student athletes revealed improvement in perceptual-motor skills following neurofeedback training aimed at enhancing SMR and beta rhythm and decreasing the theta wave, as previously reported [144,145,146,147]. Also, the effectiveness of this training in improving working memory performance (Direct and Reverse Digit Span) has been shown, consistent with the results obtained from other studies [12,148,149,150,151,152].

Suppression of the theta rhythm reduces drowsiness [147], while enhancement of the beta rhythm leads to increased concentration, sustained attention, and problem solving [153], and these effects, together with the facilitation of motor learning due to an increased SMR rhythm [80], lead to increased attention (resulting in improved working memory) and improved regulation of one’s balance through better control over the muscles that maintain posture [144]. Finally, studies by Gołaś et al. [43] and Maszczyk et al. [48] also used the TBR protocol and showed that it is effective in improving visual processing efficiency in terms of attention and reaction time, both simple and complex, in judo athletes. In this regard, see also Christie and Werthner [154]. The results of these studies are in agreement with reports in the literature that show that a suppression of the theta rhythm simultaneously with an enhancement of the beta wave is effective in improving attentional processes and reducing reaction times [155,156,157].

However, theta and beta waves can also be trained separately. For example, the study by Chen et al. [57] examined the effect of the Function-Specific Instruction (FSI) approach (based on function-directed verbal instructions that provide participants with the strategy to control the main parameters of EEG during neurofeedback) on performance in the putting task, showing a significant improvement in performance after neurofeedback of the Frontal Midline Theta wave (FMT). The frontal region is associated with top-down attentional processes [158], and an increase in FMT coincides with an increase in attentional resource allocation [159] and improvement in working memory [160], selective attention [161], and executive functions [162], so neurofeedback training aimed at increasing the FMT could result in increased attentional focus, which in turn leads to improved performance [163,164].

Finally, the study by Mikicin and colleagues [40], on the other hand, showed improved attention and focus in both the experimental group (shooting performance) and the control group following neurofeedback training aimed at beta wave enhancement. Beta waves are associated with a state of mental activity, high alertness, concentration, and focused and sustained attention, promote detail-oriented cognitive processing, and increase arousal. The results of this study are in agreement with those of other studies [165,166,167,168] and the theory of signal detection, according to which vigilance is required when a relevant stimulus appears infrequently and, when it does, immediate attention is demanded [169]. Consistent with the studies of Colloca and Benedetti [170] and Kaptchuk [171], a placebo effect was found in the control group suggesting that, even in the presence of false feedback, mere concentration on the exercise could help improve the level of attention.

In reviewing the literature on neurofeedback (NF) training in sports, it is crucial to critically examine the protocols and electrode placements used in the studies to understand the factors contributing to their effectiveness or lack thereof. The NF training protocols varied widely across the reviewed studies, which may contribute to the heterogeneity of the results. For example, some studies focused on enhancing specific frequency bands such as alpha, beta, or SMR (sensorimotor rhythm), while others utilized complex protocols targeting multiple bands simultaneously. The diversity in training protocols reflects the individualized nature of NF training, but it also complicates the comparison of results across different studies.

For instance, the study by Mikicin et al. [28] employed a protocol that amplified SMR and beta1 bands while reducing theta and beta2 bands, showing significant improvements in reaction times and performance accuracy in various sports. Conversely, Mirifar et al. [29] used a protocol to decrease theta and beta bands but did not observe significant improvements in attention and reaction time. These discrepancies suggest that the efficacy of NF training may depend on the specific frequency bands targeted and the individual characteristics of the athletes.

Electrode placement is another critical factor that can influence the outcomes of NF training. Different brain regions are associated with distinct cognitive and motor functions, and targeting the appropriate regions is essential for achieving the desired effects. However, the reviewed studies often lacked detailed descriptions of electrode placements, making it challenging to replicate their findings or draw definitive conclusions about their effectiveness.

For example, studies targeting the frontal lobes, such as the one by Liu et al. [24], aimed to improve executive functions and attention. In contrast, those targeting the sensorimotor cortex, like the study by Cheng et al. [30] on dart players, focused on enhancing motor control and reducing anxiety. The positive results of Cheng et al.’s study highlight the potential benefits of precise electrode placement for specific performance outcomes. However, the generalizability of these findings to other sports remains uncertain, particularly when considering different skill levels and contexts.

The variability in protocols and electrode placements across studies underscores the need for standardized guidelines in NF training research. While many studies report positive outcomes, the variability in results indicates that NF may not be universally effective for all athletes or sports. This variability is evident in the study by Kober et al. [56], which demonstrated significant improvements in triathletes’ performance, suggesting that athletes more experienced in self-regulation may benefit more from NF training. However, whether these results apply to other sports or less experienced athletes is still an open question.

It is important to note that the utility of neurofeedback is not universal across all sports disciplines. While some methods, like SMR training, consistently show improvement in fine motor control and accuracy, other protocols, such as theta–beta ratio training, are more suited to tasks involving complex motor skills and reaction times [43]. This suggests that neurofeedback protocols must be tailored to the specific neurocognitive demands of the sport in question.

An important aspect to consider when applying neurofeedback to athletes is the level of expertise. The benefits of neurofeedback may vary depending on the athlete’s initial skill level and stage of skill development. Studies included in this review have demonstrated that novice athletes tend to show more pronounced improvements in motor skills and reaction times, whereas elite athletes experience more subtle gains in cognitive aspects, such as attention and stress regulation. This suggests that the impact of neurofeedback may differ significantly based on the athlete’s experience level, with novices benefiting from more immediate enhancements, while experienced athletes may require more targeted and refined protocols to further improve their performance.

Future research should aim to address these inconsistencies by adopting more standardized and detailed reporting of protocols and electrode placements. Studies should include larger and more diverse samples to improve the generalizability of the findings. Comparative studies examining different NF protocols and electrode placements across various sports and skill levels would provide valuable insights into optimizing NF training for athletic performance.

## 5. Limitations and Future Directions

This section will describe the limitations concerning both the level of the review itself and the level of the articles included in it. The first limitation has to do with both these aspects and is certainly related to the selection of studies and their consequent quality assessment. In order to provide a more exhaustive description of the state of the art with regard to research on the use of neurofeedback in sport psychology between the years of 2016 and 2023, both randomized and non-randomized studies were included in this review, but many showed a medium to high risk of bias, thus introducing a greater risk of distortion of results. As for the randomized controlled trials, most did not provide information regarding the randomization process by which participants were assigned to each group, so we cannot know whether the samples studied were representative of the population (selection bias). Another bias present in most studies is detection bias due to the lack of blinding of outcome assessors to the intervention provided, which may therefore have influenced the assessment of the study results. Finally, a further critical issue found in slightly less than half of the studies relates to the comparability of groups at baseline, due to the lack of (or very little) information regarding the inclusion criteria of the participants. Moving on to the specifics of the non-randomized studies, once again, selection bias is the most present bias (none of the studies considered met this assessment criterion), and the second critical point observed relates to the failure to take into account possible confounders that might have influenced the results, mentioned in about half of the studies. These biases represent an important source of result distortion, so readers of this review should be careful in their interpretation. Future studies should attempt, as far as possible, to randomize participants into experimental and control groups and provide a clear description of the randomization method used (thus reducing the risk of selection bias), adopt a double-blind design so as to reduce the risk of detection bias, and identify clear criteria for inclusion of participants in order to obtain comparable groups. These measures should be taken to increase the reliability, reproducibility, and validity of the results, reduce the risk of bias, and improve the robustness of the evidence on the effect of neurofeedback training in the field of sports psychology.

The second limitation of the review itself concerns the lack of protocol registration, which is recommended by several guidelines in order to increase the transparency and reproducibility of a systematic review. In order to decrease potential bias, we tried to be as clear as possible about the decisions made and the methodology used. The third limitation could be related to the search strategy, as only original empirical studies were included in this review, leaving out other forms of publication (such as grey literature) that could have provided additional material for review. Finally, the fourth limitation could be the presence of a publication bias, i.e., an editorial preference to publish positive results, which leads authors not to submit studies with negative results [170,171], as only one study was found to have negative results. The presence of unpublished research could obviously have led to different results if it had been included in this review, but as no meta-analytic measures were used in this review, this is unknown.

With regard to the limitations of the studies included in this review, the second one concerns the size of the samples employed, consisting of only a few subjects each due to the difficulty in recruiting and performing controlled designs with athletes, especially elite athletes. The third limitation concerns the low number of investigations including women, as only eight studies recruited a female sample and a further six studies did not provide information regarding the gender of the participants. Future research should investigate this aspect considering the relevance and growth of the women’s sports movement. The fourth limitation found relates to the lack of a control group in some studies (which makes it impossible to determine the cause–effect relationship between the intervention and the observed outcome) and the involvement of a passive control group in others, which could lead to the occurrence of a placebo effect. The passive control groups do not undergo neurofeedback training, so the observed effects may not be due to a specificity of training in the EEG frequency bands under study. Future research should include an active control group to minimize this problem and lend more robustness to the results. A further limitation relates to the experimental context: most of the considered studies collected the athletes’ performance data within a research laboratory, while only two studies also carried out the research in the field (using the Wingate 5-Step Approach) [172]. In order to have greater reliability and ecological validity of the results, future studies should adapt their experimental protocols to field conditions (practice and competition), comparing the results obtained in this way with those obtained in the laboratory and highlighting any differences so that it can be determined whether and to what extent the results are distorted by the laboratory environment and how much this affects the outcome of the research. The sixth limitation of some of the reviewed studies relates to the lack of information regarding the sports disciplines evaluated and participants’ skill levels, which makes it difficult to provide a generalizable summary of the results. Future research should therefore be more careful in providing this sort of information in order to make the results transparent and reproducible. Likewise, further investigation is needed with open skills such as volleyball and basketball, in order to provide a more complete understanding of the effectiveness of neurofeedback in sport. Finally, most of the studies investigated the short-term effects of neurofeedback training, i.e., the results are mainly evaluated either right after the training or within a very short period following the intervention. This obviously does not make it possible to determine whether the effects of the intervention also remain stable in the medium to long term, which is why researchers should include follow-up measurements in their designs in future studies.

In addition to sport-specific limitations, this review also identifies broader issues that require attention. Firstly, the generalizability of neurofeedback protocols across different sports is uncertain. While certain protocols have proven effective in precision sports, such as shooting and golf, their applicability to team sports or endurance sports remains less explored. Further comparative studies across diverse sports disciplines are necessary to validate the broader application of neurofeedback training. Secondly, the characteristics of the athletes, including their age, gender, and skill level, may influence the effectiveness of neurofeedback interventions. Most of the studies included in this review involve small sample sizes, and only a few have focused on female athletes, making it difficult to generalize the results. More inclusive studies with diverse participants are essential to improving external validity. Thirdly, most of the studies were conducted in controlled laboratory settings, which may not fully represent the complex, high-pressure environments of actual sports competitions. To enhance the ecological validity of neurofeedback studies, future research should implement field-based studies and compare the results to those obtained in laboratory conditions.

Despite the promising results of neurofeedback in improving athletic performance, its widespread implementation raises ethical concerns regarding access. Neurofeedback requires expensive equipment and specialized professionals, making it more accessible to athletes in well-funded regions or organizations. This disparity could exacerbate inequalities in sports performance, as athletes in resource-poor settings may lack access to this technology. Future discussions should focus on developing strategies to make neurofeedback more affordable and accessible to all athletes, ensuring a level playing field and addressing potential ethical concerns.

There is ongoing debate about whether neurofeedback could be considered a form of ‘cheating’ in the context of sports. Unlike pharmacological methods, neurofeedback does not introduce foreign substances into the body but rather optimizes the athlete’s own cognitive processes. In this sense, it is comparable to traditional forms of mental and physical training. However, as neurofeedback evolves and integrates with other forms of enhancement, such as non-invasive brain stimulation, ethical concerns may arise regarding fairness and equal access [173,174,175,176]. Future research and regulatory bodies will need to consider these issues to ensure that neurofeedback and related technologies are used responsibly in competitive sports.

## 6. Conclusions

The present review aimed to provide an updated and comprehensive analysis of the latest developments in neurofeedback (NF) training within sports disciplines. By reviewing articles published between 2016 and 2023, including both randomized and non-randomized studies, this review identified significant insights into the efficacy and methodologies of NF training for athletes.

The results of this review highlight that a majority of the reviewed studies support the effectiveness of NF in enhancing both sports performance and cognitive functions in athletes. However, the diversity in the frequency bands trained and the protocols used across different studies suggests that while positive effects are often observed, the specific parameters for optimal NF training remain inconsistent and warrant further investigation.

Despite these positive findings, the review also underscores several limitations within the existing literature. These include small sample sizes, a lack of studies including female athletes, the frequent absence of control groups, and the predominance of laboratory-based research settings, which may not accurately reflect real-world conditions. Many studies did not provide sufficient detail regarding the sports disciplines evaluated or the skill levels of participants, making it difficult to generalize the findings broadly across different types of sports and levels of expertise.

Future research should focus on addressing these limitations by employing larger, more diverse samples, including both genders equally, utilizing more rigorous experimental designs with appropriate control groups, and conducting field-based studies to enhance ecological validity. Studies should aim to provide detailed reporting on the sports disciplines and participants’ skill levels to facilitate better understanding and application of NF training across various contexts. The findings suggest that neurofeedback protocols must be tailored to the specific cognitive and motor demands of the sport in question. While SMR training is beneficial for fine motor skills and accuracy, other protocols like the theta–beta ratio are better suited for complex motor tasks requiring quick reactions. Hence, standardizing sport-specific neurofeedback protocols is crucial for maximizing performance outcomes.

In conclusion, while neurofeedback training shows promise for improving athletic performance and cognitive function, a more standardized approach and rigorous methodology in future research are essential to fully harness its potential and provide more definitive guidelines for its implementation in sports.

## Figures and Tables

**Table 1:** Summary of key characteristics of included neurofeedback studies in sports.

Authors and Year	Sample	Discipline and Expertise Level	Study Design and Procedure	NF Device	Training Sessions	Electrode Position and Intervention	Feedback	Control Group	Outcome	Intervention Effect
Rijken et al., 2016 [35]	Group A: 11 professional soccer players. Group B: 10 track and field athletes (sprinters and hurdlers). Mean age not specified.	Soccer and track and field. Level: professional and élite.	Design: pilot study. No randomization (groups were not meant to compare). Procedure: pre-intervention measurements − peak performance training + biofeedback (Group A)/neurofeedback (Group B) − post-intervention measurements − follow-up measurement.	Neurofeedback system for home-training: Samsung galaxy Tab 10.1 tablet + a set of headphones (Philips, O’Neill stretch head-band); 5 Ag/AgCl electrodes mounted in the stretch headband and the ear covers of the headphone to measure EEG signals; signals transmitted via Bluetooth to the tablet (system validated by van Boxtel et al. [36]).	Group A: 6 sessions per week for 5 weeks, 3 times per day, 3 min per session. Group B: 20 sessions in 5 weeks, 30 min per session.	C3 and C4. Increase alpha power.	Auditory	No	EEG. ECG. Sleep quality. Recovery and stress. Sports Improvement Measurement-60. Performance.	Peak performance program + either HRV-feedback or neurofeedback may lead to changes in performance-related outcomes and stress reduction. Group A: EEG alpha power and LF/HF ratio improved and SIM60 emotional stability and concentration indices revealed better scores after intervention. Athletes: HRV low frequency power and recovery index of the RESTQ significantly improved.
Hosseini & Norouzi, 2017 [37]	30 volleyball players: 15 élite players and 15 non-élite players (mean age 22.8 ± 4.2, all males)	Volleyball. Level: élite and non-élite.	Design: quasi-experimental study. Procedure: pre-test phase − neurofeedback training − post-test phase.	ProComp Infiniti + BioGraph software (version 6.0)	1 single session lasting 30–45 min.	C3, C4 and T3 (International 10-10 System). Increase SMR power and inhibit alpha power.	Visual	No	Assess the use of self-talk with the Self-Talk Questionnaire (FSTQ; Theodorakis, Hatzigeorgiadis & Chroni [38]) and the correctness and precision of volleyball serve skills with the AAHPERD Volleyball Serve Test (1984)	Use of internal self- talk in elite and non-elite volleyball players significantly reduced; standard volleyball service scores significantly increased
Maszczyk et al., 2018 [39]	18 judo athletes (mean age 21 ± 1.5)	Judo. Level not specified.	Design: double-blind, randomized-controlled study. Procedure: pre-test phase– neurofeedback training − post-test phase.	Enobio wireless and portable EEG/EOG/ECG monitoring device (with bandwidth: 0 to 125 Hz and sampling rate: 500 SPS) and Neuroelectrics Instrument Controller, v 1.1 − NIC 1.1 + BioGraph Infiniti Software (version 6.0)	10 sessions of 25 min each.	O1 and O2. Inhibit theta and reinforce beta rhythms.	Visual-auditory	Yes (sham feedback)	Assess dynamic balance and EEG measures.	Theta and alpha values decreased, whereas beta values increased. Enhancement of dynamic balance.
Mikicin, Szczypińska & Skwarek, 2018 [40]	27 student-shooters (aged 19–21)	Shooting. Level: professional soldiers.	Design: randomized control study. Procedure: pre-test measurement − neurofeedback training − post-test measurement.	EEG DigiTrack Biofeedback system.	20 sessions 1/2 times a week lasting 40 min each.	F3, F4, P3 and P4. Strengthen beta frequency.	Visual	Yes (sham feedback)	Analyze changes in the level of attention and activation with COG and FLIM tests from the Vienna Test System.	Improvement of accuracy and speed in the COG test.
Norouzi et al., 2019 [41]	30 dart players (mean age 24.5 ± 4.7, all males)	Darts. Level: novice.	Design: randomized control study. Procedure: pre-test phase − neurofeedback training − retention test 1 − pressure condition − retention test 2.	Device not specified.	10 sessions of 40 min each.	F4. Suppress alpha rhythm.	Visual	Yes (mock feedback)	Assess the impact of the Quiet Mind Training on the acquisition of dart throwing skills and on the suppression of alpha power and the effect of a pressure condition on the dart throwing skills acquired under Quiet Mind Training conditions.	Improvements in implicit skill acquisition due to alpha power suppression. Stability of improvements under pressure conditions.
Szczypińska, 2019 [42]	18 handball players (mean age not specified, 9 females)	Handball. Level: 1st League and 2nd League.	Design not specified. Procedure: pre-training measurements − neurofeedback training − post-intervention measurements.	EEG DigiTrack Biofeedback system.	20 sessions 1/2 times a week lasting 40 min each.	C3 and C4. Increase beta and SMR bands and decrease theta and beta2 bands.	Visual	No	Analyze changes in peripheral vision, sensorimotor coordination and attention with PP, SMK and COG tests from the Vienna Tests System.	Improvement in concentration and attention (COG) and in sensorimotor coordination (SMK) in both males and females and in peripheral perception (PP) in males.
Mirifar et al., 2019 [31]	38 soccer players: SMR, Theta/Beta and Control group (aged 14–23, all males).	Soccer. Level not specified.	Design: mixed-multifactorial. Randomization. Procedure: pre-test 1 − pre-test 2 − neurofeedback training − post-test.	NeXus-10 MKII system + BioTrace+ software V2018A1.	10 sessions every other day for 20 days.	Cz. Theta/Beta group: decrease theta band and increase beta power. SMR group: increase SMR power.	Visual-auditory	Yes (sham feedback)	Assess concentration, selective attention and reaction times.	No improvement in attention performance and reaction times.
Gołaś et al., 2019 [43]	12 judo athletes (aged 22–25, all males)	Judo. Level: national team.	Design: randomized control study. Procedure: pre-training phase − 1st training cycle − four-week break − 2nd training cycle − post-training phase.	ProComp5 + BioGraph Infiniti software (version 6.0).	Two training cycles: 1. 15 sessions every second day lasting 4 min each. 2. 15 sessions on consecutive days lasting 4 min each.	C3. Decrease theta and beta2 bands and increase SMR and beta1 bands.	Visual-auditory	Yes (sham feedback)	Assess reaction speed.	Significant improvement in simple and complex reaction time following each training cycle. Improvement of coordination and the mechanisms of visual information processing.
Dana, Rafiee & Gholami, 2019 [44]	30 young athletes (experimental group mean age 13.26, control group mean age 12.87, all males)	Discipline not specified. Level not specified.	Design: semi-experimental study. Procedure: pre-training measurements − neurofeedback training − post-training measurements.	ProComp2 + BioGraph Infiniti software (version 6.0).	12 sessions twice a week for 6 weeks, 1 h per session.	Fz, F4, F3, O1 and Cz. Increase SMR rhythm, enhance beta band and suppress theta wave.	Auditory	Yes (passive control group)	Assess working memory performance (Wechsler digit span test) and perceptual-motor skills (Lincoln-Oseretsky test).	Improvement in working memory performance (direct and reverse digit span) and perceptual-motor skills.
Mikicin et al., 2020 [45]	7 swimmers (mean age 20.6 ± 1.40)	Swimming. Level not specified.	Design not specified. No randomization. Procedure: pre-training tests − neurofeedback training − post-training tests.	System Flex 30 + TruScan Software (version 1.1)	20 sessions for 4 months (every 7 days on average), 6 series of 5 min each per session.	C3 and C4. Decrease beta2.	Visual	No	EEG. EMG. Progressive Test. Wingate Test. Kreapelin Test.	Improved mental work performance which facilitates optimization of psychomotor activities.
Gong et al., 2020 [46]	45 student-shooters: SMR, Alpha and Control group (mean age 19.5 ± 2, all males).	Shooting. Level: University level.	Design not specified. Randomization. Procedure: pre-training measurement − neurofeedback training − post-training measurement.	Device not specified.	6 sessions in 3 weeks, 30 trials per session, 25 min per session	Cz, C3, C4 T3 and T4. SMR group: enhance SMR band in Cz, C3 and C4. Alpha group: enhance alpha band in T3 and decrease alpha band in T4.	Visual-auditory	Yes (passive control group)	Assess shooting performance.	Higher shooting performance of the SMR group. Lower shooting performance of the Alpha group. Neuroplasticity promotion.
Christie, Bertollo & Werthner, 2020 [47]	31 ice hockey players (mean age 21.7 ± 2.0, 18 females)	Ice hockey. Level: University level.	Design: longitudinal stratified random control experimental design. Procedure: two phases: adaptation phase and intervention phase + post-training assessments. Adaptation phase: 5 shooting assessments. Intervention phase: 14 shooting assessments + 15 SMR-NFT/BFT sessions.	ProComp Infiniti + BioGraph software (version 6.0).	15 sessions lasting 1.5 h each over the period of 4.5 months.	Cz. Increase SMR rhythm and inhibit theta and high beta bands.	Visual-auditory	Yes (passive control group)	Assess shooting performance.	Shooting performance improvement. Increase in SMR activity in lab setting. No changes in SMR activity during performance.
Maszczyk et al., 2020 [48]	12 judo athletes (aged 22–25, all males)	Judo. Level: national team.	Design: randomized control study. Procedure: pre-training phase − 1st training cycle − four-week break − 2nd training cycle − post-training phase	Deymed Truscan system (soft. version 6.34.1761)	Two training cycles: 1. 15 sessions every other day lasting 10 min each. 2. 15 training sessions every other day lasting 4 min each.	C3. Increase beta1 rhythm and suppress theta rhythm.	Visual-auditory	Yes (sham feedback)	Assess reaction speed.	Significant reduction in reaction time.
Domingos et al., 2020 [49]	45 participants: 15 athletes, 15 non-athletes and 15 control subjects (mean age 23.31 ± 4.20)	Discipline not specified. Level not specified.	Design: randomized control study. Procedure: Athletes: familiarization phase − pre-test phase − neurofeedback training − performance test between 6th and 7th session − post-test phase. Non-athletes: familiarization phase − pre-test phase − neurofeedback training − performance test between 5th and 6th session and 10th and 11th session − post-test phase.	Device not specified.	Athletes: 12 sessions of 25 trials of 60 s each, total time 300 min; sessions performed 2 times per week. Non-athletes: 5 blocks of trials, 5 trials of 1 min each; 25 min per session, total time 375 min.	Cz. Increase alpha power.	Visual	Yes (passive control group)	Assess short-term memory (Digit Span) and reaction time (Oddball Task) performances and standard and individual alpha bands amplitude.	Increase in SAB and IAB only in non-athlete group. Improvement in short-term memory tests in both control and athlete groups. Improvement in reaction time only in athlete group.
Shokri & Nosratabadi, 2021 [50]	45 basketball players: Group 1 biofeedback, Group 2 biofeedback + neurofeedback, Control group (mean age 25, all males)	Basketball. Level: novice.	Design: randomized control study. Procedure: pre-training assessment − neurofeedback/biofeedback training − post-training assessment.	ProComp Infiniti + BioGraph Infiniti software (version 6.0).	Group 1: 24 sessions in the lab (3 sessions per week in 8 weeks) + 8 sessions in the field. Group 2: 24 sessions (3 sessions per week in 8 weeks): 40 min biofeedback + 20 min neurofeedback per session.	Cz and Cpz. SMR protocol, increase alpha band and inhibit theta band.	Auditory	Yes (passive control group)	Assess basketball performance: free throw test, lay-up test, chest passing test and dribbling test.	Improvement in lay-up, dribbling and free throw of group 2 compared to group 1. Combined intervention more effective than biofeedback intervention alone.
Domingos et al., 2021a [51]	45 student-athletes: noisy room, silent room, control group (mean age 22.02 ± 3.05, 7 females)	Discipline not specified. Level not specified.	Design: randomized control study. Procedure: 1 familiarization session − pre-test phase − neurofeedback training − post-test phase.	Device not specified.	12 sessions of 25 trials of 60 s each, total time 300 min; sessions performed 2 times per week.	Cz. Increase IAB.	Visual	Yes (passive control group)	Assess impact of noise on working memory (N-Back Test) and reaction times (Oddball Task) and on IAB.	Both silent and noisy room had no results in increasing IAB. Significant results in all performance tests in the noisy room group.
Domingos et al., 2021b [52]	45 student-athletes: three-session-per-week intervention group, two-session-per-week intervention group, control group (mean age 21.20 ± 2.62 for the two-session protocol vs. 22.60 ± 1.12 for the three-session protocol, all males)	Discipline not specified. Level not specified.	Design: randomized control study. Procedure: 1 instruction session − pre-test phase − neurofeedback training − post-test phase.	Device not specified.	12 sessions of 25 trials of 60 s each, total time 300 min; sessions performed 2 or 3 times per week.	Cz. Improve Individual Alpha Band (IAB) amplitude.	Visual	Yes (sham feedback)	Assess changes in alpha activity and cognitive performance (Digit Span, N-Back and Oddball Task).	Better EEG results in the relative IAB amplitude in the three- compared to the two-session-per-week group. Significant improvement in N-Back and Oddball cognitive performance tests in the three-session-per-week group.
Domingos et al., 2021c [53]	30 student-athletes: three-session-per-week group, two-session-per-week group (mean age 21.20 ± 2.62 for the two-session protocol vs. 22.60 ± 1.12 for the three-session protocol, all males)	Discipline not specified. Level not specified.	Design: randomized study. Procedure: 1 instruction session − pre-test phase − neurofeedback training − post-test phase.	EEG training plugin included in the Somnium software (Cognitron, SP, Brazil).	12 sessions of 25 trials of 60 s each (EEG and HRV recordings), total time 300 min; sessions performed 2 or 3 times per week.	Cz. Improve IAB amplitude and HRV.	Visual	No	Assess if an α-NFT can increase HRV.	Significant improvement in IAB amplitude and HRV only in the three-session-per-week group.
Mottola et al., 2021 [54]	Study 1A: 40 student-athletes: increase left frontal activity group (NFL), increase right frontal activity group (NFR), passive control group (aged 18–45, 14 females). Study 1B: 26 student-athletes from Study 1A: NFL and NFR groups (9 females)	Cycling. Level not specified.	Design: randomized between-subject study (Study 1A); randomized within-subject study (Study 1B). Procedure Study 1A: visit 1 (anthropometric measurements + incremental ramp test on cycle-ergometer) − visit 2 (EEG recording + assessment of mood and self-control + brief writing task to elicit mild cognitive depletion and fatigue + second assessment of mood and self-control) − neurofeedback training − final assessment of mood and self control − cycling test on cycle-ergometer. Procedure Study 1B: visit 3 (participants received the opposite neurofeedback intervention, they received both the NFL and NFR interventions on separate occasions)	BioExplorer software (version 1.7).	1 session consisting of 6 blocks of 2 min each.	F3 and F4. NFL group: decrease F3 alpha power and increase F4 alpha power. NFR group: increase F3 alpha power and decrease F4 alpha power.	Visual-auditory	Yes (passive control group)	Assess the performance on the cycle-ergometer (time-to-exhaustion test)	Study 1A: greater relative left frontal cortical activity enhance cycling-based endurance exercise performance. Study 1B: results from Study 1A confirmed.
Wang et al., 2022 [55]	30 golf players: increased Mu rhythm group (IMG), decreased Mu rhythm group (DMG), sham group (SG) (mean age 27.4, 15 females)	Golf. Level: novice.	Design: stratified random control experimental study. Procedure: pretest phase − neurofeedback training − post-test phase.	BioTrace+ software V2018A1.	1 session lasting 30–45 min.	Cz. IMG group: increase Mu rhythm. DMG group: decrease Mu rhythm.	Auditory	Yes (sham feedback)	Assess the association between Mu rhythm and visuomotor tasks (golf putting task).	Significantly decreased Mu power in DMG group, but no significantly increased Mu power in IMG group. Significantly increased perceived control of action and improved performance in DMG group.
Kober et al., 2022 [56]	26 triathletes: real feedback group, sham feedback group (mean age 30.3, 12 females) and 25 control participants: real feedback group, sham control group (mean age 30.06, 12 females)	Triathlon. Level not specified.	Design: randomized study. Procedure: pre-training phase − neurofeedback training − post-training phase.	SIMULINK software (The MathWorks, Natick, USA).	1 session lasting 45 min.	Cz. Increase SMR rhythm.	Visual	Yes (sham feedback)	Assess self-regulation abilities and brain structure (MRI).	Real feedback groups (triathletes and controls): up-regulation of SMR power, with a stronger linear increase in the second half of the training session in triathletes. Real feedback triathletes: larger brain volumes in inferior frontal gyrus, larger grey matter volumes in right inferior frontal gyrus, increased white matter volumes bilaterally in inferior frontal gyrus, insula and orbitofrontal cortex, larger white matter volumes in left medial frontal gyrus and left precuneus. Real feedback controls: larger gray matter volumes in left inferior temporal gyrus, left parahippocampus, left fusiform gyrus and left precuneus.
Chen et al., 2022 [57]	36 golf players: function-specific instruction group (FSI), traditional instruction (TI) group, sham control group (mean age 37.1, 22 females)	Golf. Level not specified.	Design not specified. No randomization. Procedure: pre-training phase − neurofeedback training − post-training phase.	ProComp5 Infiniti + BioGraph Infiniti software (version 6.0).	1 session lasting 1.5 h divided in 2 stages: pre-NFT and acquisition.	Fz. Decrease frontal midline theta (FMT) power.	Auditory	Yes (sham feedback)	Assess performance in golf putting task.	FSI group: significant improvement in putting performance, significant decrease in 4–7 Hz power.
Mikicin & Orzechowski, 2022 [58]	10 track and field athletes and 10 swimmers (aged 18–25)	Track and field and swimming. Level not specified.	Design not specified. Procedure: pre-training measurements − neurofeedback training − post-training measurements.	System Flex 30 + TruScan software (version 2.0).	20 sessions for 4 months (every 7 days on average), 6 series of 5 min each per session.	C3 and C4. Decrease beta2 band.	Visual	Yes	Assess changes in EEG during exercise in attention states, warm-up, submaximal effort and recovery states.	Substantial modulation of spectral amplitude within sources located near frontal lobe, sensory cortex, motor cortex and anterior parietal and occipital lobes. Increased activity in sensorimotor cortex induced by submaximal exercise.
Pourbehbahani et al., 2023 [59]	40 student-golf players (mean age 26.1, 20 females)	Golf. Level: novice.	Design: randomized semi-empirical study. Procedure: pre-test phase − neurofeedback intervention − post-test phase − follow-up.	ProComp5 Infiniti + BioGraph Infiniti software (version 6.0).	6 sessions (each consisting of 20 min of neurofeedback/sham practices followed by golf putting for 3 blocks of 12 trials)	Cz. Enhance SMR wave.	Visual	Yes (sham feedback)	Examine combined effects of neurofeedback practice combined with self-control practices on motor learning (golf putting task).	Individual independent effects of neurofeedback practice and self-control practice on motor performance and learning in golf putting. No combined effect. Maintenance of positive effects in follow-up for neurofeedback training but not for self-control technique.

**Table 2:** Study quality appraisal.

Study	Screening Questions	Qualitative	Quantitative (Randomized)	Quantitative (Non-Randomized)	Quantitative (Descriptive)	Notes	Quality Score
Rijken et al., 2016 [35]	YY			NYNYN		No clear cut points for inclusion of participants. Athletes were not randomized and groups were not meant to compare. In Group B, one subject was lost at T2 and of two subjects one EEG measurement was missing because of insufficient signal quality because of woolly haired persons. The aim for each participant was to practice 20 times at home during the intervention period. A mean of 14.8 times were actually practiced. Two participants had technical problems and two participants had compliance problems. No control group existed, so causality could not be determined. It is unclear whether effects were generated because of placebo, coaching, training effects, or specific biofeedback training.	40%
Hosseini & Norouzi, 2017 [37]	YY			NYYNY		Quasi-experimental design. No randomization. No mention of confounders. Causality could not be determined due to the absence of a control group.	60%
Maszczyk et al., 2018 [39]	YY		?NYYY			No details on randomization methods, only general information. Groups not comparable at baseline.	60%
Mikicin, Szczypińska & Skwarek, 2018 [40]	YY		?NY?Y			No details on randomization methods, only general information. No information about blinding of outcome assessors. A placebo effect may have been triggered in the control group. Groups not comparable at baseline.	40%
Norouzi et al., 2019 [41]	YY		YNYYY			Groups not comparable at baseline.	80%
Szczypińska, 2019 [42]	YY			NYYNY		No randomization. Causality could not be determined due to the absence of a control group. No information about inclusion criteria of participants. No mention of confounders.	60%
Mirifar et al., 2019 [31]	YY		?YY?Y			No details on randomization methods, only general information. Of 45 participants recruited, the experiment was completed by 38 (7 were lost after the baseline measurement, before NFT intervention) for which data were complete. No information about blinding of outcome assessors.	60%
Gołaś et al., 2019 [43]	YY		?NY?Y			No details on randomization methods, only general information. No information about blinding of outcome assessors. Groups not comparable at baseline.	40%
Dana, Rafiee & Gholami, 2019 [44]	YY			NYYYY		Semi-experimental design with convenience sampling.	80%
Mikicin et al., 2020 [45]	YY			NYYNY		No randomization. Causality could not be determined due to the absence of a control group. No mention of confounders.	60%
Gong et al., 2020 [46]	YY		?NYYY			No details on randomization methods, only general information. No information about blinding of outcome assessors. Groups not comparable at baseline.	60%
Christie, Bertollo & Werthner, 2020 [47]	YY		YNN?N			19 of the original 31 participants were analyzed. One subject was eliminated due to lefthandedness, and two participants were eliminated due to trigger in light malfunction during recordings. Eight participants failed to complete the study due to Olympic commitments (n = 2), life stress (n = 1), injury (n = 3), and dropout (n = 3). Three of the eight participants in the SMR-NFT/BFT group completed fewer than 15 sessions (10 and 12 SMR-NFT/BFT sessions). No information about blinding of outcome assessors. Groups not comparable at baseline.	20%
Maszczyk et al., 2020 [48]	YY		?NY?Y			No details on randomization methods, only general information. No information about blinding of outcome assessors. Groups not comparable at baseline.	40%
Domingos et al., 2020 [49]	YY		?YY?Y			No details on randomization methods, only general information. No information about blinding of outcome assessors.	60%
Shokri & Nosratabadi, 2021 [50]	YY		?NY?Y			No details on randomization methods, only general information. No information about blinding of outcome assessors. Groups not comparable at baseline.	40%
Domingos et al., 2021a [51]	YY		?YY?Y			No details on randomization methods, only general information. No information about blinding of outcome assessors.	60%
Domingos et al., 2021b [52]	YY		?YY?Y			No details on randomization methods, only general information. No information about blinding of outcome assessors.	60%
Domingos et al., 2021c [53]	YY		?YN?Y			No details on randomization methods, only general information. Of 30 participants, 3 were excluded from the study due to poor-quality of the collected HRV data (1 from the 3 sessions/week group and 2 from the 2 sessions/week group). No information about blinding of outcome assessors.	40%
Mottola et al., 2021 [54]	YY		YNY?Y			No information about blinding of outcome assessors. Groups not comparable at baseline.	60%
Wang et al., 2022 [55]	YY		?YN?Y			No details on randomization methods, only general information. 49 trials rejected pretest and posttest (amplitudes exceeding ± 100 µV). ANOVA results indicated that differences in the number of trials didn’t affect findings. No information about blinding of outcome assessors.	40%
Kober et al., 2022 [56]	YY		?YNYY			No details on randomization methods, only general information. Two triathletes and three controls had to be excluded from the analysis because of bad EEG data quality (1 male, 4 females, too many muscle- and eye movement artifacts).	60%
Chen et al., 2022 [57]	YY			NYNYY		Consecutive sampling method. Twenty-two trials were rejected in the pre-test and 24 in the post-test because they had epochs with amplitudes exceeding ± 100 μV, which may have been contaminated by artifacts. ANOVA results indicated that differences in the number of trials didn’t affect findings.	60%
Mikicin & Orzechowski, 2022 [58]	YY			NYYNY		No randomization. No mention of confounders.	60%
Pourbehbahani et al., 2023 [59]	YY		?NY?Y			No details on randomization methods, only general information. Groups not comparable at baseline. No information about blinding of outcome assessors.	40%

Note: Y = Yes (criterion met); N = No (criterion not met); ? = Cannot tell (not enough information).

## Data Availability

No new data were created or analyzed in this study. Data sharing is not applicable to this article.
